# Authors’ Response to Peer Reviews of “Data Obfuscation Through Latent Space Projection for Privacy-Preserving AI Governance: Case Studies in Medical Diagnosis and Finance Fraud Detection”

**DOI:** 10.2196/72527

**Published:** 2025-03-12

**Authors:** Mahesh Vaijainthymala Krishnamoorthy

**Affiliations:** 1 Stelmith, LLC, 2333 Aberdeen Pl, Carollton, TX, 75007, United States, 1 9459001314

**Keywords:** privacy-preserving AI, latent space projection, data obfuscation, AI governance, machine learning privacy, differential privacy, k-anonymity, HIPAA, GDPR, compliance, data utility, privacy-utility trade-off, responsible AI, medical imaging privacy, secure data sharing, LSP, artificial intelligence

*This is the authors’ response to peer-review reports for “Data Obfuscation Through Latent Space Projection for Privacy-Preserving AI Governance: Case Studies in Medical Diagnosis and Finance Fraud Detection.”*

## Round 1 Review

### Reviewer AP [1]

#### Specific Comments

##### Major Comments

*1. What was the basis of taking up health care cancer diagnosis and financial fraud for the study [2]? Will latent space projection be an effective method for privacy protection in speech therapy to analyze audio datasets to assist in diagnosing and treating speech-related disorders; in medical imaging video datasets from endoscopy, ultrasounds, and robotic surgeries for diagnostics and artificial intelligence (AI)–assisted tools; and in telemedicine to analyze video feeds for remote consultations and diagnoses?*

**Response:** The basis for taking this up is to show data privacy through images and records for individuals. I would love to extend the research and will work on another paper for your suggestions. Thanks for the suggestion.

*2. The basic structure of the paper is missing. Please follow the guidelines of journal paper writing with distinctly visible sections of Introduction, Method, Result/Findings, Discussion, and Limitations with future scope and conclusion. The introduction, background, and related work should be written cohesively, and all should come under the Introduction heading.*

**Response:** I have revised the paper with major formatting changes and made it follow the Introduction-Methods-Results-Discussion formatting style as per the suggestion.

*3. The statistical tables are in excess. The tables and values should be talked about in written form. Limit the number of images and tables to 5‐6 or according to the journal guidelines. Use an appendix for the flowchart and any other tabular data that is too lengthy.*

**Response:** Statistical tables were reduced to only 3, and Figures are limited to 6 in total, but the flowchart is necessary inside the main paper.

*4. Explanations of tables and figures should be in paragraph form. Please cite literature where comparative inference and process-specific benefits and drawbacks are mentioned. Examples are Tables 1-5. For writing sections like “Comparative Analysis with Existing Techniques,” all the subparts should be written in paragraphs and discuss the values and analysis only, and put them in their respective paragraphs, removing the tabular data. Please use appendices for excessive tables. Within the body of the research paper, 5‐6 figures and tables are sufficient; the rest should be put in appendices.*

**Response:** Tables have been removed and converted into paragraphs

*5. In “Latency and Performance analysis, part A” and “Performance optimization” are mentions of the literature, which should be present as part of the literature in the Introduction paragraph. Restating the literature again is redundant. Stick to the structure of the journal paper. Please cite references to support the claims, such as “real-time requirements of financial systems” under the section of Real-Time Performance.*

**Response:** Thanks; moved to the Literature section and removed from there.

*6. “Scalability analysis” and other sections: What were the criteria for the choice of datasets for the study for the case studies? What were the data sizes? Give specifications in the first paragraph of respective case studies. Presenting the details about the process of procurement of files, data extraction, limitations in data handling, etc. Are there any limitations in adopting the latent space projection methods?*

**Response:** Scalability analysis was added with the source of the dataset and the data extraction and limitations. Mostly, there are a lot of advantages compared to other privacy-preserving techniques in latent space projection; the comparative analysis proves that, and a few limitations were added as well.

### Reviewer AR [3]

#### General Comments

*I thoroughly enjoyed reading this paper as it is a well-written article that will make an important contribution to the literature on the development of privacy-preserving AI governance. I have attached a few comments to improve the study.*

**Response:** Thanks for the compliment. Thanks for your time.

#### Specific Comments

##### Major Comments

*Something like a discussion that embeds the latent space projection for AI governance and the results in the current scientific debate is missing before or after Chapter VII.*

##### Minor Comments

*In Chapter II B (Existing privacy-preserving techniques), please provide some further sources to demonstrate that the challenges mentioned are still relevant, as some sources are relatively old (eg, from 2009).*

**Response:** I tried to address all your comments.

## Round 2 Review

### Reviewer AP

#### General Comments

*This paper is highly relevant to health care, particularly in the context of privacy management of data during the analysis of imagery.*

**Response:** Thanks for your time and effort. I appreciate it. Your comments were valuable. I addressed all your comments in this revision.

#### Specific Comments

##### Major Comments

*1. The case studies should be written in a more descriptive style. Please reduce the use of numbered or bullet points (in the Introduction, Method, and Result) to align with the formal writing style typically suitable for journal papers.*

**Response:** Removed all the bullets and converted most of them into paragraphs; some were aligned as paragraphs, but the bullet and numbered points were removed. The paper is in the Introduction-Methods-Results-Discussion format.

*2. Please rephrase the description of Table 3 (immediately following the table) in a narrative style. This approach enhances the readability of the article.*

**Response:** Rephrased the description for all the tables and figures, added descriptions for two other figures, explaining the figures deeply to make it more even, uniform, and readable, and for smooth flow.

*3. Two figures should not be positioned consecutively. Include some text between Figure 3 and Figure 4. Adjust and reorganize the content to ensure a smooth flow.*

**Response:** Addressed by adding content between 2 figures; now it makes it more readable and flows smoothly. Thanks.

##### Minor Comments

*4. The titles of tables and figures should be presented as captions. Revise the captions to ensure they do not begin with a verb.*

**Response:** Revised all the captions for tables and figures and made them capitalized and more readable.

Thanks for your comments.

## Abbreviations

AI: artificial intelligence

## References

[1] Singh R (2025). Peer review of “Data Obfuscation Through Latent Space Projection for Privacy-Preserving AI Governance: Case Studies in Medical Diagnosis and Finance Fraud Detection”. JMIRx Med.

[2] Vaijainthymala Krishnamoorthy M (2025). Data Obfuscation Through Latent Space Projection for Privacy-Preserving AI Governance: Case Studies in Medical Diagnosis and Finance Fraud Detection. JMIRx Med.

[3] Bommhardt T (2025). Peer review of “Data Obfuscation Through Latent Space Projection for Privacy-Preserving AI Governance: Case Studies in Medical Diagnosis and Finance Fraud Detection”. JMIRx Med.

